# Transcriptomic, Proteomic and Metabolomic Analysis of Flavonoid Biosynthesis During Fruit Maturation in *Rubus chingii Hu*

**DOI:** 10.3389/fpls.2021.706667

**Published:** 2021-08-10

**Authors:** Xiaobai Li, Jingyong Jiang, Zhen Chen, Aaron Jackson

**Affiliations:** ^1^Zhejiang Academy of Agricultural Sciences, Hangzhou, China; ^2^Taizhou Academy of Agricultural Sciences, Linhai, China; ^3^College of Life Sciences, Taizhou University, Taizhou, China; ^4^Independent Researcher, Stuttgart, AR, United States

**Keywords:** flavanol-anthocyanins, gene, enzyme, flavonoid biosynthesis, Chinese raspberry

## Abstract

*Rubus chingii* HU, is a medicinal and nutritious fruit, which is very rich in flavonoids. However, the biosynthesis of its flavonoids is poorly understood. This study examined flavonoids and the genes/proteins at four fruit ripening phases using LC-MS/MS and qPCR. Six major kinds of anthocyanins, primarily consisted of flavanol-anthocyanins, which differed in form or concentration from other *Rubus* species. In contrast to other known raspberries species, *R. chingii* had a decline in flavonoids during fruit ripening, which was due to down-regulation of genes and proteins involved in phenylpropanoid and flavonoid biosynthesis. Unexpectedly, anthocyanin also continuously decreased during fruit maturation. This suggests that anthocyanins are not responsible for the fruit’s reddish coloration. Flavanol-anthocyanins were derived from the proanthocyanidin pathway, which consumed two flavonoid units both produced through the same upstream pathway. Their presence indicates a reduction in the potential biosynthesis of anthocyanin production. Also, the constantly low expression of RchANS gene resulted in low levels of anthocyanin biosynthesis. The lack of RchF3′5′H gene/protein hindered the production of delphinidin glycosides. Flavonoids primarily comprising of quercetin/kaempferol-glycosides were predominately located at fruit epidermal-hair and placentae. The proportion of receptacle/drupelets changes with the maturity of the fruit and may be related to a decrease in the content of flavonoids per unit mass as the fruit matures. The profile and biosynthesis of *R. chingii* flavonoids are unique to *Rubus*. The unique flavonol pathways of *R. chingii* could be used to broaden the genetic diversity of raspberry cultivars and to improve their fruit quality.

## Introduction

*Rubus chingii* Hu is distributed widely across many Asian countries, such as China, Korea and Japan. It has been documented in ancient Chinese pharmacopeia including “Shen-nong-ben-cao” (Shennong’s classic of materia medica) and “Ben-cao-gang-mu” (Compendium of Materia Medica), as well as in Korean pharmacopeia. Its health benefits are believed to include improving renal function (Ding, 2011), protecting hepatocytes (Zhang et al., 2015) and relieving anxiety, pain and inflammation (Sun et al., 2013). Red raspberry (*Rubus idaeus*), black raspberry (*Rubus occidentalis*) and Chinese raspberry (*R. chingii*) all belong to subgenus Idaeobatus. The fruit of *R. chingii* is an aggregate fruit of drupelets (each containing a single seed) around the central receptacle (Chen et al., 2021). Unlike red or black raspberries, the drupelets of *R. chingii* do not detach from the receptacle at maturity. Typical phases of fruit maturation are “mature green phase (MG),” “green yellow phase (GY),” “orange yellow phase (YO),” and “red phase (RE).” During maturation, MG and GY fruits are both hard but different colors; YO fruits begin to soften and become orange; RE fruits rapidly soften and become red. The unripe fruit is traditionally used in Chinese medicine while the ripe fruit is appreciated by consumers not only for its special flavor but also for its nutritional properties.

Flavonoids occur ubiquitously in dietary and medicinal plants, which mainly consist of anthocyanins, as well as condensed and hydrolyzable tannins. These flavonoids contribute to the taste, flavor, color and pharmaceutical uses such as astringent actions. Prior studies have extensively examined *Rubus* flavonoids. For example, anthocyanin compositions have been identified and quantified in raspberries. Red and black raspberry share the same profile of anthocyanins. Their anthocyanins are predominantly cyanidin glycosides (e.g., glucosides, sophorosides, rutinosides, sambubioside, and glucosyl-rutinosides), but they only contain low to trace levels of pelargonidin glycosides (Mazur et al., 2014; Ludwig et al., 2015; Kula et al., 2016). Flavonols in red and black raspberry, as well as in Chinese raspberry, are mainly kaempferol/quercetin glycosides with the glucosides rutinoside and coumaroylglucoside (Kula et al., 2016; Yu et al., 2019). During the process of fruit ripening, the flavonoids dramatically change in composition and content, which is associated with the transformation of fruit pigmentation and flavor. In recent years, high-throughput sequencing of mRNA has been performed during the fruit ripening process in many *Rubus* species, e.g., red raspberry (*R. idaeus* cv. Nova) (Gutierrez et al., 2017), blackberry (*Rubus* spp. Var. Lochness) (Garcia-Seco et al., 2015) and black raspberry (*Rubus coreanus*) (Hyun et al., 2014; Chen et al., 2018) and (*R. occidentalis*). In red raspberry, an active anthocyanin biosynthesis takes place in the fruits during ripening (Gutierrez et al., 2017). In black raspberry (*R. coreanus*), anthocyanins and flavonols greatly increase during fruit development, while flavanols and proanthocyanidins are only accumulated at the very beginning of fruit set (Chen et al., 2018). The up-regulation of F3′H1, DFR4 and LDOX1 is responsible for the accumulation of cyanidin derivatives during the process (Hyun et al., 2014; Chen et al., 2018). These transcriptomic studies provide important information on genes in flavonoid biosynthesis.

Previous phytochemical studies mainly focused on the immature fruit in *R. chingii* (Guo et al., 2005; Ding, 2011) as only the immature fruit were used in traditional Chinese medicine. However, few studies have been conducted on *R. chingii* flavonoid biosynthesis. The purpose of this study was to investigate major flavonoid components and explore the potential mechanism underlying flavonoid biosynthesis.

## Materials and Methods

### Plant Material

**FIGURE 1:**
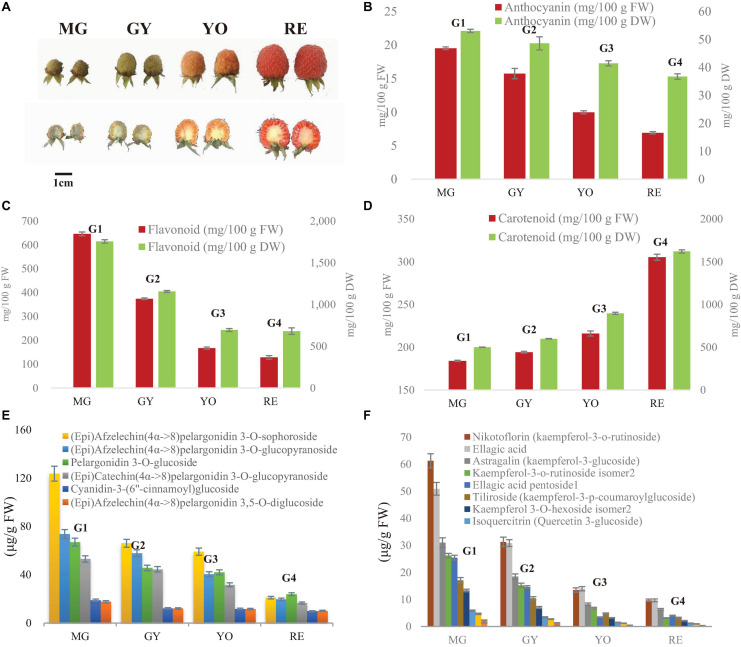
Dynamic change of fruit appearance, anthocyanins and flavonoids during maturation in *R. chingii*. **(A)** Fruit appearance, **(B)** total carotenoid, **(C)** total anthocyanin (expressed as cyanidin 3-glucoside equivalents), **(D)** total flavonoids. The four typic phases of fruit maturation are MG, mature green; GY, green yellow; YO, yellow orange; RE, red. **(E)** Content of flavanol-anthocyanins. **(F)** Content of other flavonoids. Test of between-phases effects: significant (*P* < 0.05); error bar indicates standard deviation (SD); homogeneous subsets in Duncan’s Multiple Range test: G1, G2, G3, and G4.

*Rubus chingii* plants were collected from the wild and grown in a commercial nursery located at LINHAI, Zhejiang, China (Figure 1A). The 2 year old plants were grown in 1.5 m rows. The distance between the rows was 2.0 m. Compound fertilizer (N-P_2_O_5_-K_2_O = 15–15–15) was applied to plants. Fruits were handpicked from five to six plants (2 years old) at varying maturation phases, i.e., mature green (MG), green yellow (GY), Yellow orange (YO), and Red (RE) during the growing season (May, 2019). These fruits were put into 50 ml tubes and then immediately frozen in liquid nitrogen. Three biological replicates were designed for all experiments, with each replicate comprised of 100 g of fruit. Fruit weight varied by the maturation phase. To obtain a 100g sample approximately, 12–15 fruits were used for the RE phase, 25 for the YO, 50 for the GY, and 60 fruits for the MG phase.

### Anthocyanin, Carotenoid, and Flavonoid Content

Total anthocyanin content was determined via spectrophotometry (Li et al., 2021a). The ground fruit tissue (0.3 g FW) was mixed with 10 mL 1% (*v*/*v*) HCl methanol and incubated for 24 h at room temperature in the dark. After centrifugation, supernatants were measured for absorbance at 530, 620, and 650 nm. Total anthocyanin was estimated as cyanidin-3-glucoside equivalents (mg/g FW).

Total carotenoid was determined via spectrophotometry (Li et al., 2021b). The ground fruit tissue (0.3 g FW) was mixed 10 mL extraction solution (ethanol:acetone = 1:2). The extraction was vortexed and then put in darkness for at least 30 min until the residues became colorless. The absorbance was measured at 440, 645, and 663 nm for carotenoid, and chlorophyll a/b, respectively.

Total flavonoid content was quantified by a colorimetric assay method (Li et al., 2021a). The ground fruit tissue (0.3 g FW) was mixed with 10 mL ethanol, and then centrifuged. Of supernatant, 1 mL was mixed with 2.4 mL ethanol and 0.4 mL NaNO_2_. After incubation for 6 min, the mixture was added to 0.4 mL 10% Al(NO_3_)_3_ solution. After an additional 6 min, the mixture was added to 4 mL 4% NaOH and brought to volume of 10 mL with 100% ethanol. After 15 min at room temperature the absorbance was determined at 510 nm and measured relative to a blank extraction solvent. A calibration curve was prepared using rutin solution (8–48 μg/mL). Total flavonoid content was estimated as rutin equivalent (mg/g FW).

### Fruit Anatomy and Flavonoid *in situ* DPBA Staining

Fruits were immersed and stored in FAA solution (10 formaldehyde/5 glacial acetic acid/35 ethyl alcohol) for 1 month. Radial and Transverse sections were taken and dehydrated in a graded ethanol series (20, 40, 60, 80, 95, 100, and 100% for 30 min per step) followed by paraffin infiltration and embedding using tert-butyl alcohol as an intermediate solvent. Sections of 12–14 μm were obtained using a 0.25-mm steel microtome blade on a rotary microtome and were mounted on glass slides. The mounted sections were deparaffinized and stained with Safranin O and Aniline Blue. Finally, slides were sealed with neutral balsam, observed through a light microscope (Olympus SP 350, Japan) and photographed.

The fresh fruits were separated into several parts and then embedded in medium (SCEM, Section-Lab, Hiroshima, Japan). The surface of tissues was completely covered with adhesive medium, and then immediately frozen at −20°C. The frozen samples embedded in medium were trimmed and then carefully sliced to produce 50–80 μm fresh-frozen sections using a CM1850 Cryostat (Leica microsystems, Wetzlar, Germany) set at −20°C. The sample sections were stained in a freshly prepared aqueous solution of 0.25% (w/v) 2-aminoethyl diphenylborate (DPBA) (Tokyo Chemical industry, Tokyo, Japan) and 0.00375% (v/v) Triton X-100 (Sigma-Aldrich, Shanghai, China) for at least 15 min. A Zeiss LSM880 confocal laser scanning microscope (Carl Zeiss AG, Jena, Germany) excited the sample with 30% maximum laser power at 458 nm. The fluorescence was scanned at 475–504 nm for kaempferol derivates while at 577–619 nm for quercetin derivates (Lewis et al., 2011).

### Total RNA Extraction, Library Construction, and Bioinformatic Analysis

Fruit RNAs were extracted by CTAB method (Gambino et al., 2008). Extraction buffer (2% CTAB, 2.5% PVP-40, 2 M NaCl, 100 mM Tris-HCl pH 8.0, 25 mM EDTA pH 8.0, and 2% of β-mercaptoethanol was added just before use) was prepared at 65°C in a microcentrifuge tube. The fruit tissue powder (150 mg) was added to 900 μL extraction buffer and incubated at 65°C for 10 min. An equal volume of chloroform: isoamyl alcohol (24:1 v/v) was added, vortexed, and then centrifuged at 10,000 *g* for 10 min at 4°C. The supernatant was added to chloroform:isoamyl alcohol and transferred to a new microcentrifuge tube. LiCl (3 M final concentration) was added and incubated in ice for 30 min. RNA was selectively pelleted after centrifugation at 20,000 *g* for 20 min at 4°C. The pellet was resuspended in 500 μL of SSTE buffer (10 mM Tris–HCl pH 8.0, 1 mM EDTA pH 8.0, 1% SDS, and 1 M NaCl) pre-heated at 65°C. An equal volume of chloroform:isoamyl alcohol was added, mixed and then centrifuged at 10,000 *g* for 10 min at 4°C. The supernatant was transfered to a new microcentrifuge tube and the RNA was precipitated with 0.5 volume of cold isopropanol and immediately centrifuged at 20,000 *g* for 10 min at 4°C. The pellet was washed with ethanol (70%), dried and resuspended in DEPC-water. RNA quality was tested with an Agilent 2100 Bioanalyzer (Agilent RNA 6000 Nano Kit) (Agilent, Santa Clara, CA, United States) for RNA concentration, and their purity was determined using a NanoDrop^TM^ (Thermo Fisher Scientific, Wilmington, DE, United States).

The libraries construction followed the method described in Li et al. (2013). The mRNAs were isolated from total RNA with oligo(dT) and then fragmented. The first and second strand of cDNA were synthesized, purified and resolved with EB buffer for end repair and adenine (A) addition. After that, the cDNA fragments were connected with adapters and those with suitable size were PCR amplified. Agilent 2100 Bioanaylzer and ABI StepOnePlus Real-Time PCR System (Thermo Fisher Scientific, Rockford, IL, United States) were used to quantify and qualify the libraries.

The read data were processed following the procedure (Li et al., 2013). The low-quality reads (>20% of the bases with low quality < 10) and reads with adaptors and unknown bases (*N* > 5%) were filtered to get clean reads. The clean reads were assembled into unigenes using Trinity, for functional annotation and expression estimation. Data are available via NCBI with accession (PRJNA671545). The relative expression was estimated by Fragments Per Kilobase of transcript per Million mapped reads (FPKM). Based on the relative expression, differential expressed unigenes were defined by threshold (fold Change > 2.00 or <0.5; adjusted *P*-value < 0.05) and they were subjected to pathway enrichment (FDR < 0.01 are defined as significant enrichment).

### Real-Time Quantitative PCR Assay

The expressions of gene involved in flavonoid biosynthesis was determined by qPCR (Li et al., 2015). The reverse transcription reaction was performed with M-MLV (Takara, China), and the reverse-transcribed products were used as the template for qPCR with gene-specific primers. All reactions were assayed in three biological and technical replications, and performed in an ABI PRISM 7900HT (Applied Biosystems, United States) using Platinum SYBR Green qPCR SuperMix-UDG (Invitrogen, United States). PCR conditions consisted of: pre-denaturation and hot start Taq activation at 95°C for 5 min, then 40 cycles of 95°C for 15 s, and 60°C for 30 s. Actin was used as reference gene. The relative expression was calculated on the basis of 2^–ΔΔCt^.

### Protein Extraction, HPLC Fractionation, LC-MS/MS Assay and Bioinformatic Analysis

Fruit proteins were extracted by the method described by Li et al. (2019, 2020). The ground fruit tissue was mixed with four volumes of lysis buffer (8 M urea, 1% Triton-100, 10 mM dithiothreitol, and 1% Protease Inhibitor Cocktail), followed by sonication three times on ice. Debris were removed by centrifugation at 20,000 *g* at 4°C for 10 min. Protein was precipitated with cold 20% trichloroacetic acid (TCA) for 2 h at −20°C and then centrifugated at 12,000 *g* 4°C for 10 min. After the supernatant was discarded, the remaining precipitate was washed three times with cold acetone. The protein was re-dissolved in 8 M urea and the protein concentration was determined using a BCA protein assay kit (Thermo Fisher Scientific, Wilmington, DE, United States) according to the manufacturer’s instructions. The extracted proteins were reduced and alkylated, and then digested by trypsin.

After trypsin digestion, peptide was desalted by Strata X C18 SPE column (Phenomenex, Tianjin, China) and vacuum-dried. Peptide was reconstituted in 0.5 M TEAB and processed according to the manufacturer’s protocol for TMT kit. One unit of TMT reagent was thawed at room temperature and reconstituted in anhydrous acetonitrile (enough for about 100 μg protein). 41 μL of the TMT Label Reagent was added to each 100 μL sample and samples were labeled with different TMT tags. The peptide mixtures were then incubated for 2 h at room temperature, pooled, desalted and dried by vacuum centrifugation.

The labeled peptides were fractionated by HPLC, and the peptides were divided into nine fractions. The peptides were loaded into tandem mass spectrometry (MS/MS), Q Exactive HF-X^TM^ (Thermo Fisher Scientific, Rockford, IL, United States). These processes were performed as described by Li et al. (2019, 2020). The relative expression of protein was estimated by comparing the intensities of the reporter ions. Compared to the expression profile at the MG phase, differential expressed proteins were defined by threshold change (change fold > 1.5 or <0.67 and *P* < 0.05).

The resulting MS/MS data were processed using the MaxQuant search engine (v.1.5.2.8). Tandem mass spectra were searched against a local database of *R. chingii* transcriptome and concatenated with a reverse decoy database. Trypsin/P was used as a cleavage enzyme allowing up to 2 missing cleavages. The mass tolerance for precursor ions was set as 20 ppm in the first search and 5 ppm in the main search and the mass tolerance for fragment ions was set as 0.02 Da. Carbamidomethyl on Cys was specified as fixed modification and oxidation on Met were specified as variable modifications. FDR was adjusted to <1%. Data are available via ProteomeXchange with identifier (PXD021977).

### Analysis of Major Anthocyanins and Flavonoids

Anthocyanins were extracted with 1% (v/v) HCl methanol and concentrated by CentriVap refrigerated Centrifugal Concentrators at 8°C (Models 73100 Series) (Labconco, Kansas City, MO, United States) and then re-dissolved in 1 mL 1% (v/v) HCl methanol. Flavonoids were extracted with 70% methanol for 2 h at room temperature in the dark, concentrated with refrigerated Centrifugal Concentrators at 8°C (Labconco Models 73100 Series) and then re-dissolved in 1 mL 70% methanol. The extract was passed through a 0.22-μm microporous membrane filter for LC-ESI-MS analysis.

For anthocyanins, the mobile phases were 1% formic acid-water (A) and acetonitrile (B). Gradient conditions were as follows: 0–25 min, 5–35% phase B; 25–37 min; 35–95% phase B. The loading volume was 5 μL, the flow rate was 0.4 mL min^–1^; the column temperature was 50°C, and the UV detector was set at 530 nm. For flavonoids, the mobile phases were 0.1% formic acid-water (A) and 0.1% formic acid-acetonitrile (B). The linear gradient programs were as follows, 0–5 min, 5–10% phase B; 5–25 min, 10–25% phase B; 25–37 min, 25–95% phase B; Sample injection volume was 5 μL; Column oven temperature was 50°C; flow rate was 0.3 mL min^–1^; and the UV detector was set at 360 nm. Anthocyanins and flavonoids separated by UPLC were analyzed using a MS AB Triple TOF 5600^*plus*^ System (AB SCIEX, Framingham, MA, United States) in both negative ion (source voltage at −4.5 kV, and source temperature at 550°C) and positive ion mode (source voltage at +5.5 kV, and source temperature at 600°C). Maximum allowed error was set to ±5 ppm. Declustering potential (DP), 100 V; collision energy (CE), 10 V. For MS/MS acquisition mode, the parameters were almost the same except that the collision energy (CE) was set at 40 ± 20 V, ion release delay (IRD) at 67 and the ion release width (IRW) at 25. The IDA-based auto-MS^2^ was performed on the 8 most intense metabolite ions in a cycle of full scan (1 s). The scan range of m/z of precursor ion and product ion were set as 100–2,000 Da and 50–2,000 Da. The exact mass calibration was performed automatically before each analysis employing the Automated Calibration Delivery System.

### Statistical Analysis

The averages and standard deviations were calculated in IBM SPSS Statistics 22. The treatments were compared using a two-way analysis of variance, *T*-test and Duncan multiple Test in IBM SPSS Statistics 22. Unless otherwise stated, significant differences were set at the threshold (*p* < 0.05). KEGG enrichment of proteins were determined by a two-tailed Fisher’s exact test. The significant threshold was set up (*p*-value < 0.05) for KEGG enrichment.

## Results

### Anthocyanins and Flavonoids Composition, Anatomical Structure and Flavonoid Staining

In our previous study on *R. chingii*, anthocyanin unexpectedly decreased as fruit matured, as well as flavonoids, while carotenoids increased (Li et al., 2021a) (Figures 1B–D). Anthocyanins were mainly flavanol-anthocyanins consisting of two flavonoid units (Li et al., 2021a) (Supplementary Figure 1). Other flavonoids consisted primarily of glycosides of quercetin and kaempferol. These anthocyanins and flavonoids all significantly decreased in content during fruit ripening (Figures 1E,F). In this study, anthocyanin and flavonoid showed a similar trend of decreasing during fruit maturation (based on dry weight), while carotenoids increased (Figures 1B–D). The water content continuously increased as fruit maturated (Table 1). This suggests that decreases of anthocyanin and flavonoid during fruit ripening is probably due to down-regulation of their biosynthesis rather than an increase of water content.

**TABLE 1:** Change of water content during fruit ripening.

Phase	*Fresh weight (g) ± *SD*	*Dry weight (g) ± *SD*	*Water content% ± *SD*
MG	1.06^a^ ± 0.06	0.39^a^ ± 0.02	63.67^a^ ± 0.27
GY	1.39^b^ ± 0.08	0.45^b^ ± 0.02	67.63^b^ ± 0.35
YO	1.91^c^ ± 0.10	0.46^c^ ± 0.03	75.92^c^ ± 0.25
RE	4.78^d^ ± 0.15	0.90^d^ ± 0.06	81.11^d^ ± 0.54

*MG, mature green; GY, green yellow; YO, yellow orange; RE, red.*

DW, dry weight; FW, fresh weight.

*Significant (*P* < 0.05) in test of between-phases effects: Error bar indicates standard deviation (SD); Homogeneous subsets in Duncan’s Multiple Range test: a, b, c, and d.

**FIGURE 2:**
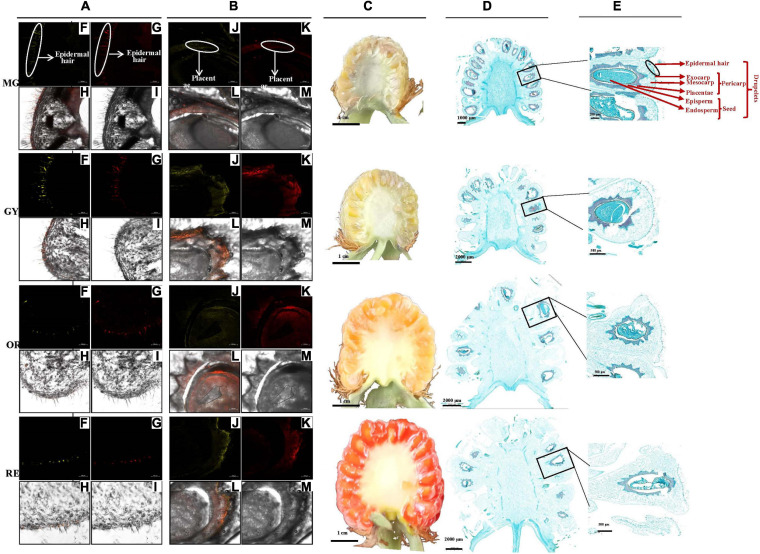
Fruit section and *in situ* flavonoid staining at four maturation phases (MG to GY, YO, and RE) (Li et al., 2021a, b). **(A,B)** Pericarp and seed radial sections by frozen method. **(C)** Fruit radial sections by scalpel and **(D,E)** by paraffin method. Fluorescence was collected at **(F,J)** 475–504 nm for kaempferol and **(G,K)** 577–619 nm for quercetin after fresh-fruit section were stained with diphenylboric acid 2-aminoethylester (DPBA). **(H,L)** Flavonoid localization in inflorescences combing **(F,J)** and **(G,K)**, and **(I,M)** original figure without fluorescence. Flavonoid mainly accumulated in epidermal hair and placentae. The proportion of different tissues gradually changed with the maturity of the fruit.

A raspberry fruit is an aggregate fruit composed of drupelets (Li et al., 2021a, b) (Figure 2). Each drupelet contains the pericarp and seed. The pericarp is made up of the exocarp, hypodermis, and mesocarp layers; while the seed consists of the episperm, endosperm, and embryo. The exocarp is attached with a layer of epidermal hair and the seed is surrounded by placentae. In fruit cross-sections, DPBA fluorescence showed flavonoid accumulation patterns at various stages of fruit maturation (Figure 2B). Flavonol-specific fluorescence was mainly observed in the fruit epidermal hair throughout the entire fruit maturation process, but rarely in fruit flesh including the exocarp, hypodermis and mesocarp (Figures 2F–M). As fruit matured, fruit epidermal hair became shorter and thinner. In addition, flavonol-specific fluorescence was seen in the placentae and seed coats of developing seed (Figures 2C–F) and the receptacle enlarged, which made up a larger proportion of the fruit than drupelets (including placentae and seed coats). Thus, the proportion of epidermal hairs and placentae (containing most of the flavonols) decrease with the maturity of the fruit, which is probably one of main reasons for a decrease in the content of flavonoids per unit mass.

### Profiling of Genes and Proteins Involved in Flavonoid Synthesis

Twelve transcriptomics were developed for MG, GY, YO, and RE fruits (three replicates for each fruit phase). A total of 89,188 unigenes were obtained, and 49,755 (55.79%) and 37,833 (42.42%) were annotated in the non-redundant and KEGG database, respectively. The biggest difference in gene expression was between RE/MG (6,502 up-regulated and 5733 down-regulated unigenes) while the smallest difference was between GY/MG (1,965 up-regulated and 1,966 down-regulated unigenes) (Supplementary Figure 2A). Accordingly, in twelve proteomes of MG, GY, YO, and RE fruits, 141,036 unique peptides corresponding to 9,478 proteins, and 8,529 quantified proteins were identified. In proteomics, 506 up-regulated and 618 down-regulated proteins were observed between RE/MG while 765 up-regulated and 799 down-regulated proteins were between GY/MG (Supplementary Figure 2B). The results suggest that the biggest difference is between RE and MG while the smallest difference is between GY and MG.

Generally, flavonoid products are involved in four pathways, i.e., phenylpropanoid biosynthesis, flavonoid biosynthesis, flavone and flavonol biosynthesis, and anthocyanin biosynthesis. KEGG enrichment was performed to discern the multivariate pattern of up- and down-regulated unigenes/proteins. The unigenes involved in “phenylpropanoid biosynthesis” and “flavonoid biosynthesis” were significantly enriched in GY/MG, YO/MG, and RE/MG and most of them were down-regulated (Supplementary Figure 3A). Accordingly, the down-regulated proteins involved in these pathways’ biosynthesis were enriched as well (Supplementary Figure 3B). However, neither up-regulated or down-regulated unigenes/proteins were enriched in “flavone and flavonol”, or “anthocyanin” biosynthesis (Supplementary Figure 3). This suggests that “phenylpropanoid biosynthesis” and “flavonoid biosynthesis” are more active in green phases than the other three phases, and responsible for biosynthesis of major flavonoid products during fruit maturation.

### Differentially Expressed Genes and Proteins in Flavonoid and Phenylpropanoid Biosynthesis

**FIGURE 3:**
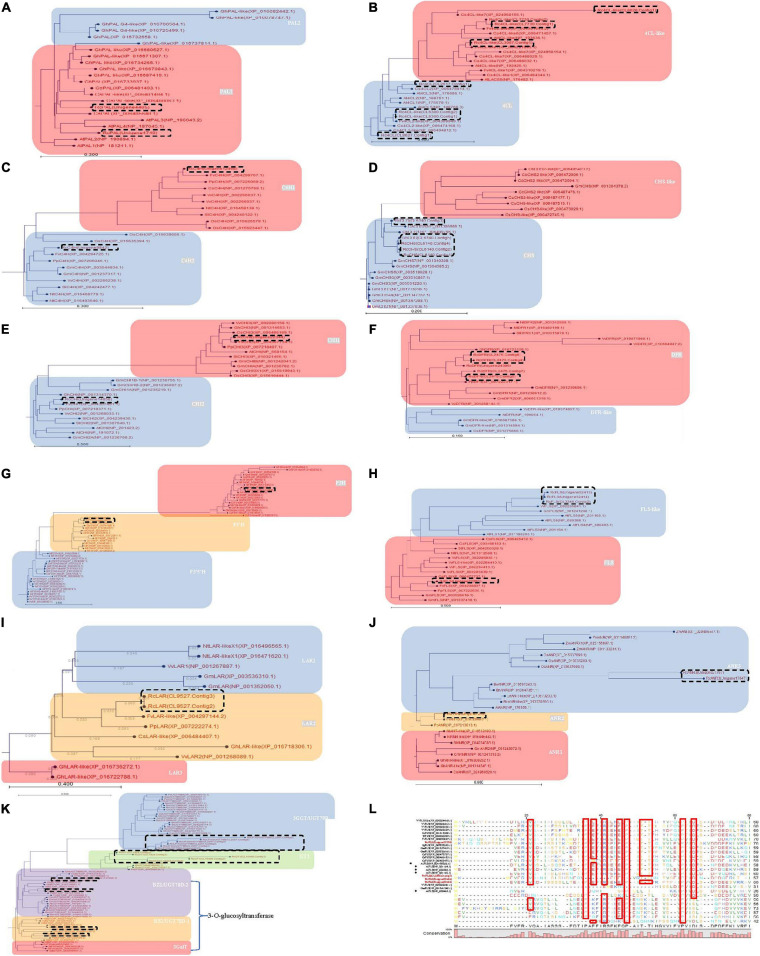
Phylogenetic analysis of the differently expressed genes involved in phenylpropanoid, flavonoid and anthocyanin biosynthesis during four maturation phases in *R. chingii*. Neighbor-Joining with 1,000 bootstrap replicates was used to construct the phylogenetic tree. The distance between deduced proteins was measured with the “Kimura Protein” method. **(A)** Phe ammonia lyase (PAL), **(B)** 4-coumaroyl-CoA synthase (4CL), **(C)** cinnamate-4-hydroxylase (C4H), **(D)** chalcone synthase (CHS), **(E)** chalcone isomerase (CHI), **(F)** bifunctional dihydroflavonol 4-Reductase/flavanone 4-Reductase (DFR), **(G)** flavanone-3β-hydroxylase (F3H), flavonoid-3′-hydroxylase (F3′H), flavonoid-3′,5′-hydroxylase (F3′5′H), **(H)** flavonol synthase (FLS), **(I)** leucoanthocyanidin reductase (LAR) **(J)** anthocyanidin reductase (ANR), **(K)** glucosyltransferase protein. **(L)** alignment of RcFLS in bold with other functionally characterized plant FLS. The residues framed by red boxes are strictly conserved in the various enzyme subclasses. *Arabidopsis* AtFLS (NP_001190266.1) marked by squares having strictly conserved residues while AtFLS (NP_201164.1), AtFLS (NP_680388.1), AtFLS (NP_201165.1), and AtFLS (NP_680463.1) are marked by diamonds and has altered or missing conserved residues. Previous studies indicate that only AtFLS1 (NP_001190266.1) encodes a catalytically competent protein and is the only member of this group that influences flavonoid levels (Owens et al., 2015). Genes in dash-line box were from *R. chingii.* Homologs in gray were down-regulated at the gene/protein level and responsible for the decrease of flavonoids as the fruit matured.

**FIGURE 4:**
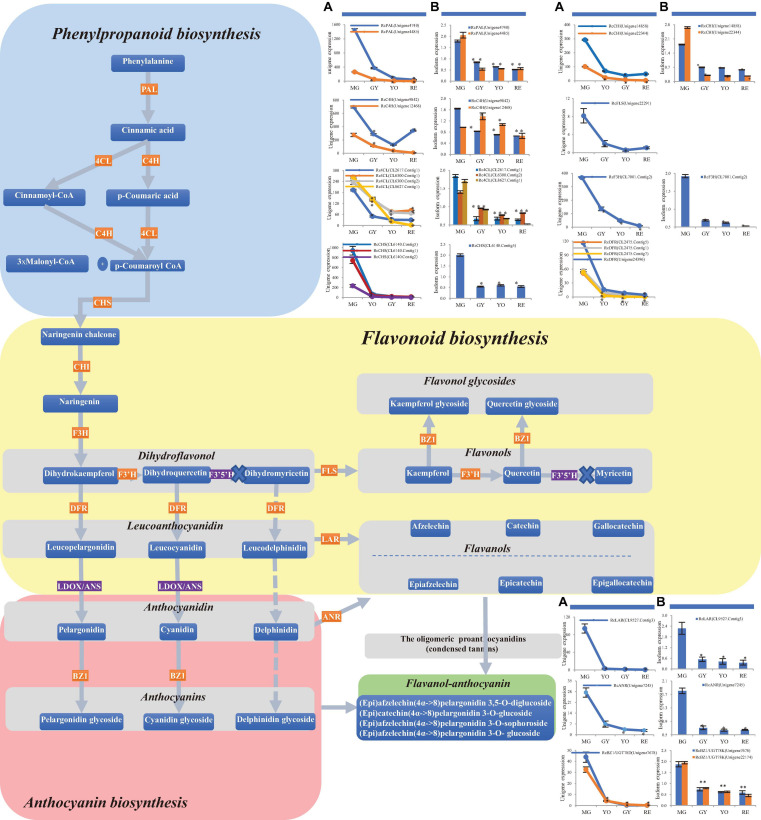
Changes in phenylpropanoid, flavonoid and anthocyanin biosynthesis during four maturation phases in *R. chingii*. **(A)** The change of mRNA unigene expression, **(B)** the change of protein expression. RcANS, shown in the purple rectangle, was expressed at a low gene level while RcF3′5′H was not detected at the gene/protein level. No expression of RcF3′5′H, shown in the purple rectangle, blocked biosynthesis of the dihydromyricetin, leucodelphinidin, (epi) gallocatechin and their corresponding glycosides. Flavanols and anthocyanins were condensed into flavanol-anthocyanins, shown in the green rectangle.

In phenylpropanoid biosynthesis, two Phe ammonia lyase (PAL) homologs, RchPAL (Unigene4740 and Unigene4485), were phylogenetically grouped together (Figure 3A) and significantly down-regulated at the gene/protein level during maturation (Table 2). Rch4CL and Rch4CL-like homologs were phylogenetically grouped into two branches, respectively (Figure 3B). The 4-coumaroyl-CoA synthase (4CL) homologs were significantly down-regulated at the gene/protein level during maturation. Rch4CL-like homologs (CL7730.Contig2, CL3087.Contig1, and 8828.Contig1) had low gene expression and their proteins were not detected (Table 2). Two cinnamate-4-hydroxylase (C4H) homologs, RchC4H (Unigene9842, and Unigene12468), were phylogenetically grouped into two different branches, i.e., C4H1 and C4H2, respectively (Figure 3C), but they had a trend of down-regulation at the gene/protein level during maturation (Table 2). Chalcone synthase (CHS) homologs were grouped together (Figure 3D) and down-regulated at the gene/protein level during maturation (Table 2). These up-stream genes/proteins were down-regulated to decrease conversion from phenylpropanoids to flavonoids (Figures 4A,B).

**TABLE 2:** The temporal change in expression of mRNA unigenes an proteins involved in phenylpropanoid, flavonoid, and anthocyanin biosynthesis during four maturation phases in *Rubus chingii* Hu.

	mRNA unigenes				Proteins			
		
	MG	GY	YO	RE	MG	GY	YO	RE
	Mean ± *SD*	Mean ± *SD*	Mean ± *SD*	Mean ± *SD*	Mean ± *SD*	Mean ± *SD*	Mean ± SD	Mean ± SD
RchPAL(Unigene4740)	1448 ± 38.35	372.9 ± 21.61*	82.76 ± 2.74*	43.93 ± 1.25*	1.79 ± 0.04	0.84 ± 0.02*	0.64 ± 0.01*	0.52 ± 0.01*
RchPAL(Unigene4485)	261.8 ± 2.12	52.63 ± 6.36*	9.16 ± 1.74*	3.25 ± 0.2*	2.05 ± 0.13	0.54 ± 0.04*	0.56 ± 0.01*	0.56 ± 0.04*
Rch4CL(CL2617.Contig1)	193.5 ± 4.78	49.16 ± 3.54*	31.24 ± 1.51*	30.05 ± 1.34*	1.85 ± 0.04	0.67 ± 0.05*	0.67 ± 0.04*	0.64 ± 0.02*
Rch4CL(CL6300.Contig1)	264 ± 4.91	138.9 ± 2.67*	74.06 ± 1.42*	81.89 ± 1.34*				
Rch4CL(CL6300.Contig2)	235.5 ± 11.2	143.7 ± 10.47*	70.4 ± 2.52*	67.14 ± 3.21*	1.41 ± 0.03	0.96 ± 0.01*	0.77 ± 0.01*	0.82 ± 0.01*
Rch4CL(CL8627.Contig1)	263.4 ± 4.6	143.2 ± 2.01*	20.65 ± 1.35*	1.75 ± 0.43*	1.71 ± 0.04	0.92 ± 0.01*	0.67 ± 0.01*	0.48 ± 0.04*
Rch4CL-like(CL7730.Contig2)	3.65 ± 0.48	1.14 ± 0.24*	0.42 ± 0.12*	0.04 ± 0.04*				
Rch4CL-like(CL3087.Contig1)	1.82 ± 0.08	6.02 ± 0.32*	5.49 ± 0.49*	0.86 ± 0.24*				
Rch4CL-like(8828.Contig1)	3.28 ± 0.19	3.85 ± 0.62	4 ± 0.17	6.62 ± 0.85				
RchC4H(Unigene9842)	692.5 ± 12.81	284.2 ± 10.64*	124.2 ± 3.25*	338.8 ± 6.83*	1.64 ± 0.02	0.83 ± 0.01*	0.71 ± 0.01*	0.67 ± 0.01*
RchC4H(Unigene12468)	274.9 ± 25.84	109.3 ± 6.21*	38 ± 0.76*	7.18 ± 0.53*	1.51 ± 0.01	1.36 ± 0.12*	1.07 ± 0.03*	0.67 ± 0.09*
RchCHS(CL6140.Contig1)	740.4 ± 56.75	43.19 ± 7.71*	21.58 ± 1.69*	13.36 ± 0.73*				
RchCHS(CL6140.Contig2)	234.5 ± 36.02	18.67 ± 7.56*	4.24 ± 0.22*	1.69 ± 1.24*				
RchCHS(CL6140.Contig4)	238.6 ± 26.79	25.21 ± 3.14*	4.08 ± 0.82*	1.61 ± 0.7*				
RchCHS(CL6140.Contig5)	938.4 ± 87.82	68.39 ± 19.58*	21.93 ± 1.95*	12.82 ± 0.19*	2.01 ± 0.06	0.55 ± 0.02*	0.61 ± 0.03*	0.55 ± 0.04*
RchCHI(Unigene14858)	293 ± 3.94	69.41 ± 3.83*	37.66 ± 0.29*	48.17 ± 1.53*	1.82 ± 0.02	0.69 ± 0.02*	0.67 ± 0.01*	0.57 ± 0.01*
RchCHI(Unigene22344)	100.8 ± 1.58	22.08 ± 0.32*	6.96 ± 0.66*	2.18 ± 0.31*	2.66 ± 0.04	0.30 ± 0.01*	0.27 ± 0.03*	0.28 ± 0.00*
RchDFR(Unigene24396)	116.2 ± 6.36	15.79 ± 5.04*	8.31 ± 0.48*	4.45 ± 0.06*				
RchDFR(CL2475.Contig1)	56.67 ± 2.72	9.29 ± 1.79*	4.39 ± 0.7*	1.26 ± 0.03*				
RchDFR(CL2475.Contig4)	41.22 ± 2.74	4.93 ± 0.88*	3.12 ± 0.05*	0.99 ± 0.13*				
RchDFR(CL2475.Contig5)	50.94 ± 0.36	3.44 ± 0.22*	0.59 ± 0.13*	0.36 ± 0.11*				
RchDFR(CL2475.Contig7)	53.15 ± 1.39	2.54 ± 0.18*	0.52 ± 0.08*	0.11 ± 0.05*				
RchF3H(CL7001.Contig2)	367.1 ± 2.13	136 ± 14.02*	48.14 ± 3.59*	9.98 ± 0.74*	1.92 ± 0.05	0.69 ± 0.02	0.62 ± 0.02	0.50 ± 0.03
RchF3′H(Unigene19522)	10.76 ± 0.7	1.67 ± 0.63*	0.15 ± 0*	2.53 ± 0.28*				
RchFLS(Unigene22291)	8.17 ± 1.6	1.98 ± 0.69*	0.57 ± 0.29*	1.07 ± 0.23*				
RchFLS-like(Unigene52413)	2.96 ± 0.16	5.17 ± 0.66	5.44 ± 3.03	3.37 ± 0.18				
RchFLS-like(Unigene52414)	4.99 ± 0.78	5.03 ± 0.17	8.87 ± 2.34	6.96 ± 0.54				
RchFLS-like(CL3345.Contig2)	3.56 ± 0.56	3.95 ± 0.05	5.89 ± 0.35	4.63 ± 0.72				
RchANR2(Unigene7245)	27.39 ± 3.01	6.86 ± 2.01*	3.39 ± 0.05*	2.67 ± 0.65*	1.81 ± 0.08	0.71 ± 0.05*	0.64 ± 0.03*	0.65 ± 0.02*
RchANR3(Unigene17647)	0.1 ± 0.1	1.47 ± 0.06*	1.68 ± 0.02*	0.06 ± 0.06				
RchANR3(Unigene27757)	0 ± 0	0 ± 0	0.43 ± 0.43	0 ± 0				
RchLAR(CL9527.Contig2)	0.36 ± 0.36	1.01 ± 0.58	0.12 ± 0.12	0 ± 0				
RchLAR(CL9527.Contig3)	93.98 ± 10.29	3.06 ± 1.18*	1.53 ± 0.17*	0.41 ± 0.06*	2.28 ± 0.34	0.55 ± 0.12*	0.42 ± 0.15*	0.36 ± 0.13*
RchANS/LODX(Unigene7480)	0.79 ± 0.2	3.41 ± 0.21*	0 ± 0	5.82 ± 0.49*				
RchBZ1/UGT78D-1(Unigene7678)	43.82 ± 4.66	4.17 ± 0.65*	0.12 ± 0.06*	0.03 ± 0.03*	1.87 ± 0.12	0.73 ± 0.07	0.61 ± 0.03*	0.59 ± 0.08*
RchBZ1/UGT78D-1(Unigene22174)	32.5 ± 2.41	4.21 ± 0*	0.84 ± 0.09*	0.11 ± 0*	1.95 ± 0.05	0.79 ± 0.03*	0.62 ± 0.03*	0.46 ± 0.05*
RchBZ1/UGT78D-2(Unigene7056)	2.79 ± 0.01	3.52 ± 0.05	3.9 ± 0.36	0.3 ± 0.03	1.10 ± 0.02	1.14 ± 0.09	1.00 ± 0.03	0.84 ± 0.03
RchBZ1/UGT78D-2(CL3164.Contig1)	8.11 ± 0.48	12.61 ± 0.73	22.92 ± 1.19*	16.59 ± 0.19*	0.81 ± 0.09	1.05 ± 0.05	1.25 ± 0.10*	1.02 ± 0.06
Rch3GGT/UGT79B(CL2207.Contig1)	0.18 ± 0	0.55 ± 0.32	0.09 ± 0.09	0.05 ± 0.05				
Rch3GGT/UGT79B(CL2207.Contig2)	2.92 ± 0.47	4.83 ± 1.47	1.4 ± 0.06	0.01 ± 0.01				
Rch3GGT/UGT79B(CL2207.Contig3)	6.2 ± 0.38	6.46 ± 0.47	5.06 ± 0.13	0.42 ± 0.24	1.09 ± 0.08	1.22 ± 0.04	0.94 ± 0.08	0.81 ± 0.04
Rch3GGT/UGT79B(CL2207.Contig4)	0.72 ± 0.01	1.41 ± 0.04	0.57 ± 0.02	0.13 ± 0.08				
Rch3GGT/UGT79B(CL2207.Contig5)	0.8 ± 0.15	0.35 ± 0.14	0.79 ± 0.14	0.88 ± 0.05				
Rch3GGT/UGT79B(Unigene4033)	0.97 ± 0.21	1.11 ± 0.23	0.25 ± 0.09	0.49 ± 0.16				
Rch3GGT/UGT79B(Unigene4034)	1.68 ± 0.04	2.71 ± 0.59	1.17 ± 0.01	0.23 ± 0.08				
RchGT1(CL10466.Contig1)	7.05 ± 0.11	6.82 ± 0.59	4.53 ± 1.22	0.6 ± 0.45				
RchGT1(CL10466.Contig3)	9.96 ± 0.72	7.4 ± 0.54	6.96 ± 0.42	9.9 ± 1.47				
RchGT1(CL1924.Contig1)	21.29 ± 0.12	68.02 ± 5.49*	86.88 ± 2.18*	53.47 ± 2.1*	0.77 ± 0.03	1.10 ± 0.03	1.13 ± 0.04	1.10 ± 0.01
RchGT1(CL1924.Contig3)	2.08 ± 0.05	1.4 ± 0.23	0.58 ± 0.06	1.59 ± 0.09				
RchGT1(Unigene268)	1.89 ± 0.05	1.07 ± 0.25	1.15 ± 0.25	0.82 ± 0.09				

*Significant difference with fold Change > 2.00 or <0.5 and adjusted *P-*value < 0.05 for unigenes; with fold > 1.5 or <0.67 and *P* < 0.05 for proteins.

*Different homologs were designated with different serial numbers, e.g., two homologs, RcPAL(Unigene4740) and RcPAL(Unigene4485).*

In flavonoid biosynthesis, chalcone isomerase (CHI) homologs were phylogenetically separated into two different branches, i.e., CHI1 and CHI2, respectively (Figure 3E), and both of them were down-regulated at the gene/protein level during maturation (Table 2). Five bifunctional dihydroflavonol 4-Reductase/flavanone 4-Reductase (DFR) unigenes, belonging to one DFR homolog, were phylogenetically grouped into the DFR branch (Figure 3F) and significantly down-regulated during maturation (their proteins were not detected) (Table 2). Flavanone-3β-hydroxylase (F3H) and flavonoid-3′-hydroxylase (F3′H) homologs were phylogenetically grouped into the F3H and F3′H branch, respectively, but neither flavonoid-3′,5′-hydroxylase (F3′5′H) unigene nor protein was detected (Figure 3G). RchF3H (CL7001.Contig2) was significantly down-regulated at the gene/protein level during maturation, while the RchF3′H (Unigene19522) unigene was maintained at a low level and its protein was not detected (Table 2). Flavonol synthase (FLS) homologs were, respectively, separated into FLS and FLS-like groups (Figure 3H). The FLS-like group was represented by FLS paralogs without a conserved functional domain (Figure 3L). FLS-like homologs were all maintained at low gene expression and none of their proteins were detected. However, RchFLS (Unigene22291) showed a decreasing trend of gene expression but remained expressed at low levels (Table 2). These genes/proteins were down-regulated, reducing the biosynthesis of flavone, flavonol, and their derivates (Figures 4A,B). Notably, a deficiency of RchF3′5 ′H blocked the conversion from dihydroquercetin to dihydromyricetin, resulting in the absence of leucodelphinidin, myricetin, gallocatechin, and delphinidin glycoside.

In anthocyanin biosynthesis, the leucoanthocyanidin dioxygenase (ANS/LODX) homolog, RchANS/LODX (Unigene7480), was maintained at low gene expression levels and its protein was not detected (Table 2). Leucoanthocyanidin reductase (LAR), RchLAR (CL9527.Contig3), and anthocyanidin reductase (ANR), RchANR2 (Unigene7245), were both significantly down-regulated at the gene/protein level during maturation (Table 2 and Figures 3I,J). In contrast, RchANR3 (Unigene17647 and Unigene27757) had very low gene expression and their corresponding proteins were not detected. Three main class of glucosyltransferase were identified, i.e., anthocyanidin 3-*O*-glucosyltransferase (BZ1/UGT78D), anthocyanidin 3-*O*-glucoside 2′′-*O*-glucosyltransferase (3GGT/UGT79) and anthocyanidin 5,3-*O* glucosyltransferase (GT1). A phylogenetic tree grouped these glucosyltransferase homologs into three main branches (Figure 3K). Two homologs, BZ1/UGT78D-1 and BZ1/UGT78D-2, had different patterns of expression (Table 2). RchBZ1/UGT78D-1 (Unigene7678 and Unigene22174) were significantly down-regulated at the gene/protein level as fruit matured, while RchBZ1/UGT78D-2 (Unigene7056) remained constantly expressed at both the gene/protein level and the RchBZ1/UGT78D-2 (CL3164.Contig1) gene/protein was slightly up-regulated. There were three homologs for each Rch3GGT/UGT79 and RchGT1, but they had relatively low expression and did not show a clear trend of change at the gene/protein level. The consistently low level of expression of RchANS suggests a relatively low concentration of anthocyanins. Notably, the genes/proteins of RchLAR and RchANR2 were highly expressed in unripe fruit, resulting in the relative abundance of flavan-3-ols, i.e., (epi) catechin and (epi) afzelechin (Figures 4A,B). These flavan-3-ols interacted with each other or with cyanin/pelargonin to generate proanthocyanins or dimeric anthocyanins, respectively.

**FIGURE 5:**
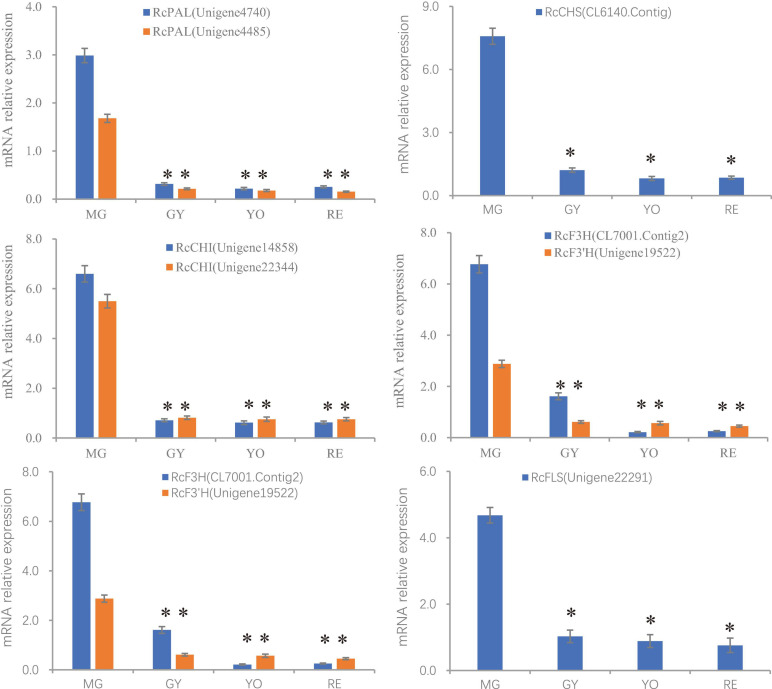
The relative expression of genes (PAL, CHS, CHI, F3H, F3′H, and FLS) involved in flavonoid production during the process of fruit maturation in *R. chingii.* The expression was estimated by real-time PCR. Actin gene was inner control. ^∗^indicates comparison between other phases and MG, T-Test, significant (*P* < 0.05).

In conclusion, most of the differently expressed unigenes and proteins in these pathways shared a similar trend of change in expression, which was consistent with the high correlations seen between them (Pearson correlation = 0.956). Additionally, the expression of gene and proteins was validated by qPCR (Figure 5 and Supplementary Table 1). However, the phylogenetically different homologs showed different patterns of gene/protein expression. The results suggest the changes observed in gene expression are consistent with those seen in protein expression, and the homologs are divergent in function and expression.

## Discussion

### Changes in Flavonoids and Their Localization During Fruit Maturation

Unexpectedly, the total anthocyanins and flavonoids both showed a continuous decrease during the fruit maturation process in *R. chingii*. This pattern was different from any previous report in *Rubus* species including red and black raspberry. In red raspberry, anthocyanin concentration continuously increases throughout fruit ripening, but the flavonoid concentration decreases before veraison stage, and then increases until maturity (Wang et al., 2009). Cyanidin glycosides are the most prominent kind of anthocyanins while quercetin glycosides are constantly present at low concentrations (Stavang et al., 2015). In black raspberry, quercetin glycosides and cyanidin glycosides both increase during ripening, while the content of flavanols and proanthocyanidin dimers decrease (Hyun et al., 2014). Ubiquitously, anthocyanins increase while flavonoids first decrease and then increase during maturation in many berries (Vvedenskaya and Vorsa, 2004; Giribaldi et al., 2007; Song et al., 2015; Li et al., 2019). The increased flavonoids after veraison is mainly due to the substantial increase of anthocyanin concentration (Li et al., 2019). However, the increased anthocyanins were not observed during fruit ripening in *R. chingii*. The continuous down-regulation of anthocyanins was responsible for the continuous decrease of total flavonoids.

Interestingly, our previous studies showed that the content of total carotenoids increased during fruit ripening (Li et al., 2021a, b). Of them, β-citraurin and its esters, was predominant and quickly accumulated at the late stage of fruit maturation. This indicates that its red coloration is not caused by anthocyanins. β-citraurin is also a color-imparting pigment involved in the reddish color of citrus fruits (Ma et al., 2013). Flavonoid *in situ* staining shows that the flavonoids (i.e., kaempferol and quercetin derivates) predominately accumulate at the same tissues (epidermal hair and placentae) of fruits (Li et al., 2021a). It is highly likely that these flavonoids are synthesized in the cells in which they accumulate. The flavonoids in epidermal hair might function as antioxidants that protect fruit from pests and pathogens, while the ones in seed may function as endogenous regulators of auxin transport that are responsible for seed maturation. As the fruit matured the epidermal hairs became thinner and shorter and many of them fell off (Figure 3C) while the receptacle became greatly enlarged makes up a relatively large proportion of the overall fruit (Figure 3D). Thus, the proportion of different tissues changes with the maturity of the fruit, which may be one of the main reasons for a decrease in the content of flavonoids per unit mass. Rather than following the regular flavonol pathway in other Rubus (up-regulation during maturation), *R. chingii* utilizes a carotene pathway producing a high level of B-citraurin as its predominant pigment.

### Down-Regulated Expression of Genes/Proteins in the Phenylpropanoid Pathway Caused a Decrease in Flux From Phenylpropanoids to Flavonoids

The genes and enzymes involved with phenylpropanoid biosynthesis and the flavonoid biosynthesis have been extensively studied in many plants. Most of these genes are involved in multigene families. Some members are divergent in function and others are redundant or underutilized (Kim et al., 2004). In *Arabidopsis*, two redundant PAL genes (AtPAL1 and AtPAL2) are both expressed in vascular tissues. AtPAL3 is primarily expressed in roots and leaves, albeit at low levels, while AtPAL4 is mainly expressed in developing seed tissue (Raes et al., 2003). These divergent PAL genes respond differentially under various developmental events and environmental stresses (Kumar and Ellis, 2001; Cochrane et al., 2004; Chang et al., 2008). In tomato, only one PAL transcript is induced by pathogen or wounding (Chang et al., 2008). In red raspberry, RiPAL1 is expressed during early fruit ripening, while RiPAL2 is expressed at later stages of flower and fruit development (Kumar and Ellis, 2001). PAL genes also show tissue specific patterns of expression. The expression of RiPAL1 transcripts is much higher than that of RiPAL2 in leaves, shoots, roots, young fruits, and ripe fruits. In blueberry, three PAL genes are up-regulated at the gene/protein level as fruit matures (Li et al., 2019). In this study, two phylogenetically close RchPALs were both down-regulated at the gene/protein level as fruit matured (Figures 4A,B).

4CL isoenzymes exhibit distinct substrate affinities due to their different metabolic functions. In *Arabidopsis*, four 4CL genes are divergent in functions, e.g., At4CL4 exhibits the rare property of activating sinapate and other 4CL substrates (e.g., 4-coumarate, caffeate, and ferulate) (Hamberger and Hahlbrock, 2004). In *Physcomitrella patens*, three 4CLs display the highest catalytic efficiency toward 4-coumarate, which is distinguished from the fourth 4CL (Silber et al., 2008). In blueberry, Vc4CL and Vc4CL-like are both up-regulated as fruit maturates although they are phylogenetically separated (Li et al., 2019). In this study, two phylogenetically distant CL4 homologs (RchCL4 and Rch4CL-like) showed distinct patterns of expression (Table 2). 4CLs were significantly down-regulated at the gene/protein level as fruit maturated while 4CL-like genes were expressed at low levels. The result suggests that 4CL rather than 4CL-like functions in down-regulation of the phenylpropanoid pathway in fruit.

C4H belongs to a large group of cytochrome P450 monooxygenases (P450) in plants and exclusively constitute the CYP73 family, a typical group of P450. In citrus, C4H1 and C4H2 are different in both expression patterns and N-termini, suggesting they have specific functions in organelles (Betz et al., 2001). In blueberry, two phylogenetically close C4H homologs (VcC4H2A and VcC4H2B) are dramatically up-regulated from the pink to blue phase (Li et al., 2019). In this study, two phylogenetically related RchC4H homologs (Unigene9842 and Unigene12468) both had down-regulated expression.

### Regulation of Genes/Proteins in Flavonoid Biosynthesis Are Responsible for the Diversity in Flavonoid Composition and Concentration

Different CHS genes are associated with the different phenotypes. In genus *Ipomoea*, six CHS genes are regulated by developmental signals. Of these, CHSD and CHSE function in flavonoid biosynthesis, especially CHSD which has dominant effects on floral pigmentation (Clegg et al., 2000). In blueberry, three phylogenetically close CHS genes share a similar pattern of up-regulation (Li et al., 2019). In Korean black raspberry, two CHS genes are both up-regulated during fruit maturation (Hyun et al., 2014). However, in *R. chingii*, the opposite occurs and one of the RchCHS is down-regulated at the gene/protein level during fruit maturation. Chalcone isomerase (CHI) is a rate-determining enzyme in flavonoid biosynthesis. In red-fruited tomato the CHI gene is expressed at low levels and decreases upon ripening while an accompanying accumulation of the CHI substrate, naringenin chalcone, occurs (Bovy et al., 2002). Heterologous expression of petunia CHI gene in tomato results in up to 70-fold increase in flavonols in the fruit peel, and a decrease in naringenin chalcone (Muir et al., 2001). In grape, CHI gene expression gradually decreases with ripening, and later, slightly increases (Wang et al., 2012). In Korean black raspberry, three CHI genes were all up-regulated during fruit maturation (Hyun et al., 2014). In *R. chingii*, two classes of RchCHI (Figure 3E) were both down-regulated at the gene/protein level during fruit maturation (Figure 4).

F3H, F3′H, F3′5′H and FLS play an important role in the types and quantities of flavonoid biosynthesis, which determines the colors and flavors of fruits (Li et al., 2019). In blueberry, F3H, F3′5′H and FLS are all up-regulated at the gene/protein level during fruit maturation (Li et al., 2019). In Korean black raspberry, two F3H genes are both up-regulated during fruit maturation (Hyun et al., 2014). In contrast, RchF3H was down-regulated at the gene/protein level during fruit maturation. Moreover, the gene expression of RchF3′H, and RchFLS were low and down-regulated during ripening. Interestingly, RchF3′5′H was absent in *R. chingii*. The results suggest that the low expression or down-regulation of these genes/proteins reduces biosynthesis of dihydroflavonol and flavonols, while a deficiency of RchF3′5′H impeded biosynthesis of myricetin and delphinidin glycoside.

DFR is responsible for branch flux from dihydroflavonol into anthocyanin while ANR and LAR converts anthocyanidin leucoanthocyanidins to flavan-3-ols and then to proanthocyanidins (condensed tannins). DFR in Korean black raspberry (Hyun et al., 2014) and blueberry (Li et al., 2019) are both up-regulated at the gene/protein level as the fruit matured. In contrast, two classes of RchDFR genes were both significantly down-regulated during fruit maturation. In blueberry, LAR protein is up-regulated during fruit maturation (Li et al., 2019). The opposite occurred in *R. chingii* with down regulation of RchLAR at the gene/protein level during fruit maturation (Table 2 and Figure 4). The results indicate that the down-regulated expression of RchDFR is responsible for the decrease of leucoanthocyanidins while the down-regulated expression of RchLAR and RchANR is responsible for the decrease of flavanols. However, the flavanols, i.e., (epi) catechin and (epi) afzelechin could be combined with pelargonin for production of dimeric anthocyanins. Notably, the biosynthesis of dimeric anthocyanins requires two flavonoid units, rather than a single flavonoid unit as is needed for monomeric anthocyanins. One unit is produced from anthocyanin biosynthesis while the other is from flavanols biosynthesis, but both share a common upstream pathway. This indicates a reduction in the potential biosynthesis of these dimeric anthocyanins. Also, the constantly low expression of RchANS causes a reduction in overall anthocyanin biosynthesis.

Flavonoid glycosyltransferases have roughly four different functional classes including 3-*O*, 5-*O*, 7-*O* glycosyltransferases and diglycoside/disaccharide chain glycosyltransferases, respectively. 3-*O* glycosyltransferase includes AtUGT78D1 and AtUGT78D2 in *Arabidopsis* (Kim et al., 2012), and CsUGT78A14 and CsUGT78A15 in *Camellia sinensis*, which are responsible for biosynthesis of flavonol 3-*O*-glucosides/galactosides, respectively (Cui et al., 2016). 5-*O* glycosyltransferase includes AtUGT75C1 (anthocyanins 5-*O* glucosyltransferase) in *Arabidopsis* (Gachon et al., 2005), and CsUGT75L12 (flavonoid 5-*O* glycosyltransferases) (Dai et al., 2017). 7-*O* glycosyltransferase includes AtGT-2 (flavonoid 7-*O*-glucosyltransferase) in *Arabidopsis* (Kim et al., 2006), and GmIF7GT (UDP-glucose:isoflavone 7-*O*-glucosyltransferase) (Noguchi et al., 2007). Moreover, there is another class of flavonoid glycosyltransferases, i.e., flavonol 3-*O*-glycoside: 2′′-*O* glucosyltransferase (3GGT/UGT79), an enzyme responsible for the terminal modification of pollen-specific flavonols (Knoch et al., 2018). In blueberry, two 3-*O* glycosyltransferase genes are both up-regulated as fruit matured, while another 5-*O* glycosyltransferase is down-regulated (Li et al., 2019). In Korean black raspberry four 3-*O* glycosyltransferase genes are all up-regulated as fruit matured. In *R. chingii*, two 3-*O* glycosyltransferases (RchBZ1/UGT78D) were all down-regulated at the gene/protein level as the fruit matured, while 5-*O* glycosyltransferase and flavonol 3-*O*-glycoside: 2′′-*O* glucosyltransferase genes were mostly maintained at low expression levels. The results indicate that the down-regulation of 3-*O* glycosyltransferase is responsible for the decreased content of flavonol glycosides (e.g., kaempferol-3-*O*-glucoside and quercetin 3-*O*-glucoside). A diversity in flavonoid glycosyltransferases leads to a variety of flavonoid glycosides, e.g., flavonoid coumaroylglucoside, flavonoid rutinoside, flavonoid sophoroside etc.

## Conclusion

In *R. chingii*, most flavonoids were located in the fruit epidermal-hair and placentae. In most berries there is an increase in the total flavonoid and anthocyanin concentration near the end of the fruit maturation. However, in *R. chingii* the unripe (mature green) fruit had much higher flavonoid levels, as well as anthocyanin concentrations, than was seen in latter phases of fruit development. The decreases of flavonoid and anthocyanin concentrations in latter phases of *R. chingii* fruit development is due to the down-regulation of phenylpropanoid, and flavonoid biosynthesis. Notably, most of anthocyanins were in flavanol-anthocyanin condensed forms, which is produced from the proanthocyanidin pathway. The mechanisms of flavonoid biosynthesis appear to be unique to *R. chingii*, and have not been reported in other fruit crops. Multiple genes and proteins in these pathways were divergent in function and differently regulated.

## Data Availability Statement

The original contributions generated for this study are publicly available. This data can be found here: transcriptomic data are available via NCBI with accession (PRJNA671545). Proteomic data are available via ProteomeXchange with identifier (PXD021977).

## Author Contributions

XL conceived of the study, performed the experiments, and wrote the manuscript. ZC analyzed mRNA expression. JJ planted *R. chingii* and collected fruit tissues. AJ assisted with writing and revising the manuscript. All authors read and approved the manuscript.

## Conflict of Interest

The authors declare that the research was conducted in the absence of any commercial or financial relationships that could be construed as a potential conflict of interest.

## Publisher’s Note

All claims expressed in this article are solely those of the authors and do not necessarily represent those of their affiliated organizations, or those of the publisher, the editors and the reviewers. Any product that may be evaluated in this article, or claim that may be made by its manufacturer, is not guaranteed or endorsed by the publisher.

## Supplementary Material

Supplementary Figure 1 Composition of anthocyanins and flavonoids in *R. chingii* (Li et al., 2021a). The upper graph(s): electrospray product ion mass spectra; the lower graph(s): fragment ions mass spectra; shown to the right of the graphs: putative molecular structure and cleavage pattern.

Supplementary Figure 2 Summary of differentially expressed **(A)** genes and **(B)** proteins during four maturation phases in *R. chingii*.

Supplementary Figure 3 KEGG enrichment of the differentially expressed **(A)** unigenes and **(B)** protein isoforms. Bar size represents the number of genes enriched in a particular pathway. Up-regulated proteins between two phases are marked in red; down-regulated proteins between two phases are marked in blue. The biosynthesis of phenylpropanoid and flavonoid are shown in rectangles.

Supplementary Table 1 Primers for RT-qPCR analysis.
