# Supplementary material for: Transcriptomic, Proteomic and Metabolomic Analysis of Flavonoid Biosynthesis During Fruit Maturation in Rubus chingii Hu
Source: Front Plant Sci. 2021 Aug 10;12:706667. doi: 10.3389/fpls.2021.706667 (PMC8384110; doi:10.3389/fpls.2021.706667)
Supplement: Supplementary Figure 1 — Composition of anthocyanins and flavonoids in R. chingii (Li et al., 2021a). The upper graph(s): electrospray product ion mass spectra; the lower graph(s): fragment ions mass spectra; shown to the right of the graphs: putative molecular structure and cleavage pattern. [file Presentation_1.PPTX]

## Slide 1
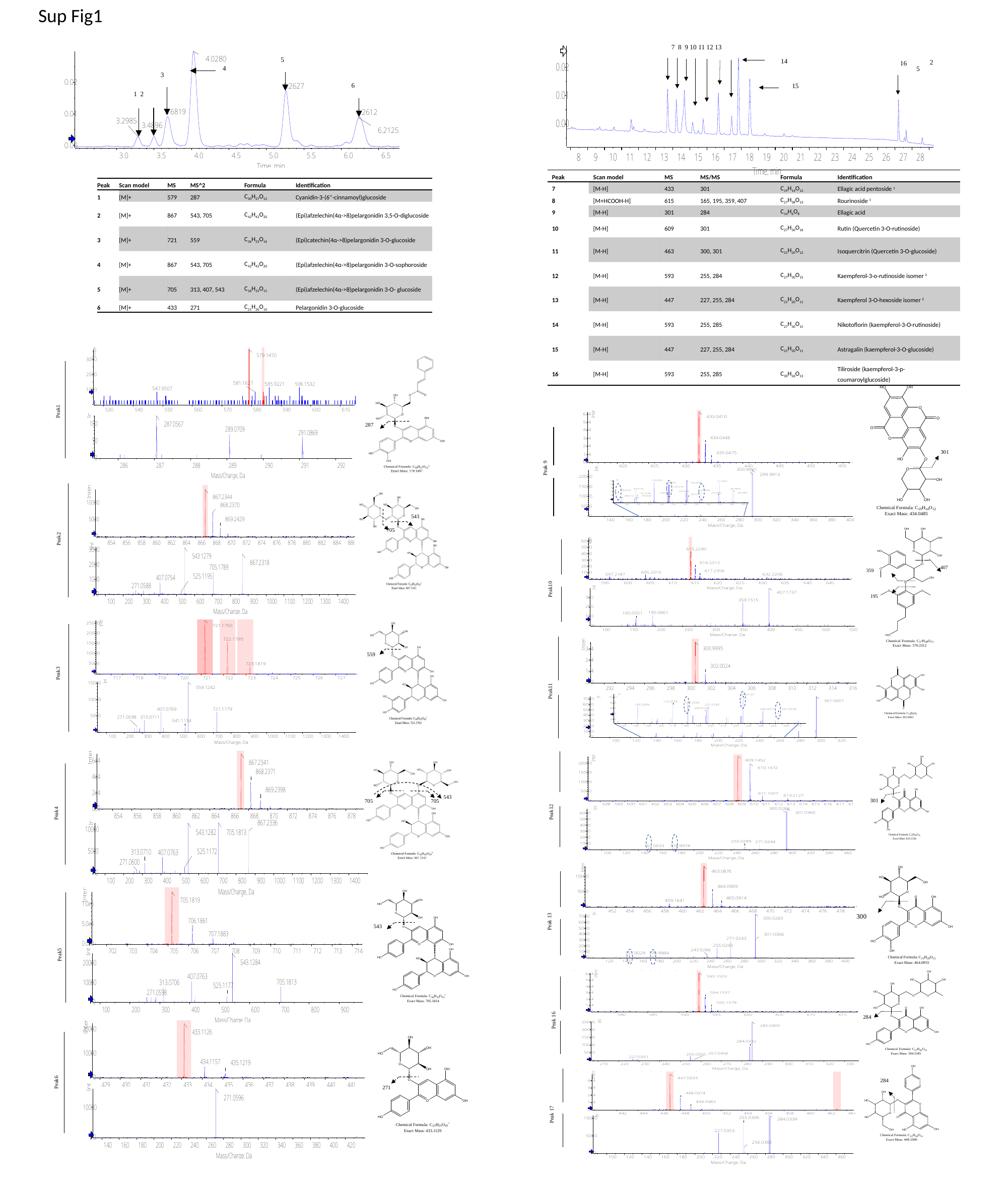

Sup Fig1
7 8 9 10 11 12 13
14
25
16
15
5
4
3
6
1 2
287
Peak1
301
Peak 9
Peak2
705
543
407
195
359
Peak10
Peak3
559
Peak11
Peak12
301
543
705
705
Peak4
300
Peak 13
543
Peak5
284
Peak 16
Peak6
271
284
Peak 17
| Peak | Scan model | MS | MS/MS | Formula | Identification |
| --- | --- | --- | --- | --- | --- |
| 7 | [M-H] | 433 | 301 | C19H14O12 | Ellagic acid pentoside 1 |
| 8 | [M+HCOOH-H] | 615 | 165, 195, 359, 407 | C27H38O13 | Rourinoside 1 |
| 9 | [M-H] | 301 | 284 | C14H6O8 | Ellagic acid |
| 10 | [M-H] | 609 | 301 | C27H30O16 | Rutin (Quercetin 3-O-rutinoside) |
| 11 | [M-H] | 463 | 300, 301 | C21H20O12 | Isoquercitrin (Quercetin 3-O-glucoside) |
| 12 | [M-H] | 593 | 255, 284 | C27H30O15 | Kaempferol-3-o-rutinoside isomer 2 |
| 13 | [M-H] | 447 | 227, 255, 284 | C21H20O11 | Kaempferol 3-O-hexoside isomer 2 |
| 14 | [M-H] | 593 | 255, 285 | C27H30O15 | Nikotoflorin (kaempferol-3-O-rutinoside) |
| 15 | [M-H] | 447 | 227, 255, 284 | C21H20O11 | Astragalin (kaempferol-3-O-glucoside) |
| 16 | [M-H] | 593 | 255, 285 | C30H26O13 | Tiliroside (kaempferol-3-p-coumaroylglucoside) |
| Peak | Scan model | MS | MS^2 | Formula | Identification |
| --- | --- | --- | --- | --- | --- |
| 1 | [M]+ | 579 | 287 | C30H27O12 | Cyanidin-3-(6''-cinnamoyl)glucoside |
| 2 | [M]+ | 867 | 543, 705 | C42H43O20 | (Epi)afzelechin(4α->8)pelargonidin 3,5-O-diglucoside |
| 3 | [M]+ | 721 | 559 | C36H33O16 | (Epi)catechin(4α->8)pelargonidin 3-O-glucoside |
| 4 | [M]+ | 867 | 543, 705 | C42H43O20 | (Epi)afzelechin(4α->8)pelargonidin 3-O-sophoroside |
| 5 | [M]+ | 705 | 313, 407, 543 | C36H33O15 | (Epi)afzelechin(4α->8)pelargonidin 3-O- glucoside |
| 6 | [M]+ | 433 | 271 | C21H20O10 | Pelargonidin 3-O-glucoside |
