# Supplementary material for: Transcriptomic, Proteomic and Metabolomic Analysis of Flavonoid Biosynthesis During Fruit Maturation in Rubus chingii Hu
Source: Front Plant Sci. 2021 Aug 10;12:706667. doi: 10.3389/fpls.2021.706667 (PMC8384110; doi:10.3389/fpls.2021.706667)
Supplement: Supplementary Figure 2 — Summary of differentially expressed (A) genes and (B) proteins during four maturation phases in R. chingii. [file Presentation_2.PPTX]

## Slide 1
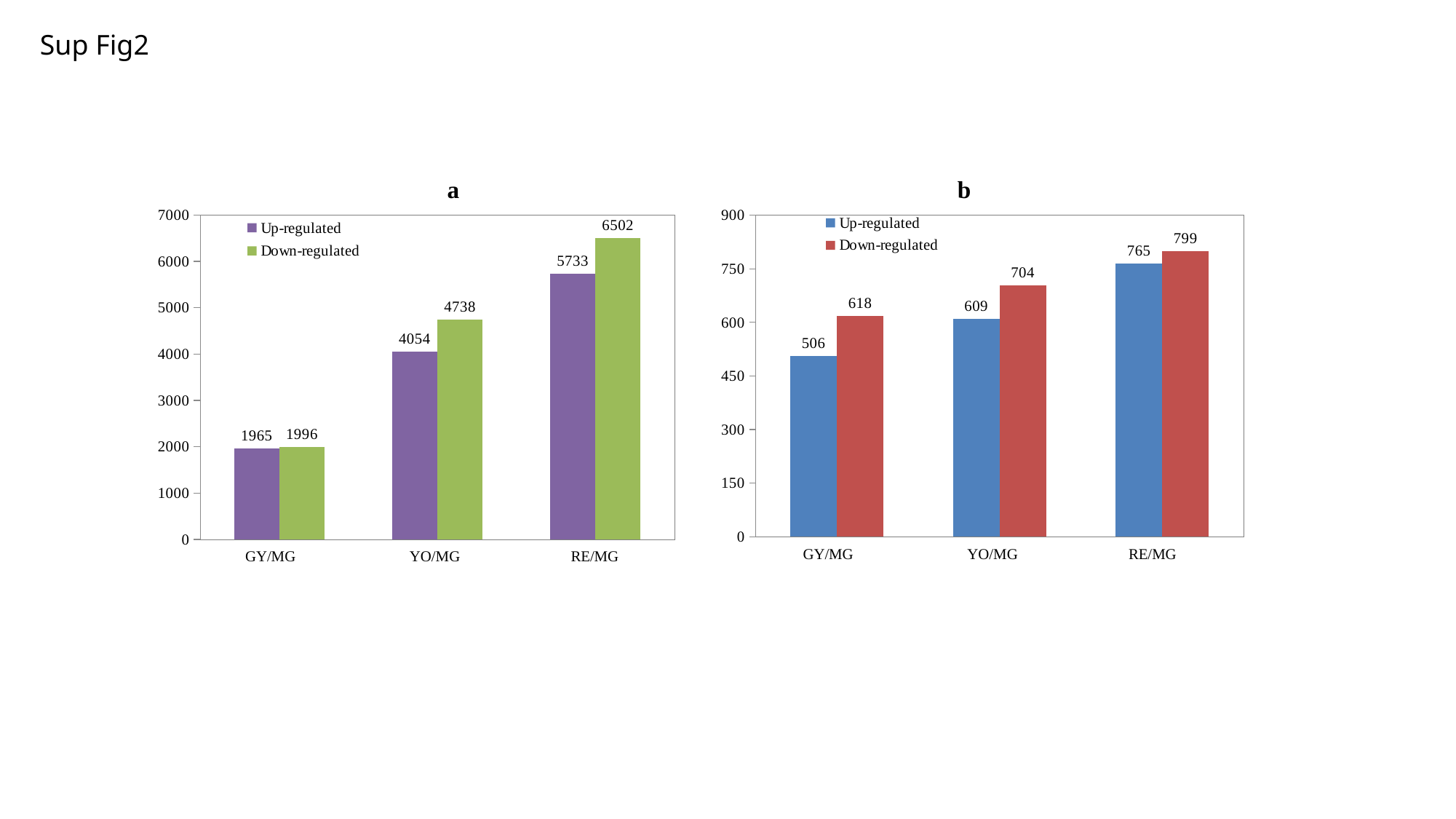

Sup Fig2
a b
### Chart
| Category | Up-regulated | Down-regulated |
|---|---|---|
| GY/MG | 1965.0 | 1996.0 |
| GY/MG | 4054.0 | 4738.0 |
| RE/MG | 5733.0 | 6502.0 |GY/MG YO/MG RE/MG
### Chart
| Category | Up-regulated | Down-regulated |
|---|---|---|
| GY/MG | 506.0 | 618.0 |
| GY/MG | 609.0 | 704.0 |
| RE/MG | 765.0 | 799.0 |GY/MG YO/MG RE/MG
