# Supplementary material for: Transcriptomic, Proteomic and Metabolomic Analysis of Flavonoid Biosynthesis During Fruit Maturation in Rubus chingii Hu
Source: Front Plant Sci. 2021 Aug 10;12:706667. doi: 10.3389/fpls.2021.706667 (PMC8384110; doi:10.3389/fpls.2021.706667)
Supplement: Supplementary Figure 3 — KEGG enrichment of the differentially expressed (A) unigenes and (B) protein isoforms. Bar size represents the number of genes enriched in a particular pathway. Up-regulated proteins between two phases are marked in red; down-regulated proteins between two phases are marked in blue. The biosynthesis of phenylpropanoid and flavonoid are shown in rectangles. [file Presentation_3.PPTX]

## Slide 1
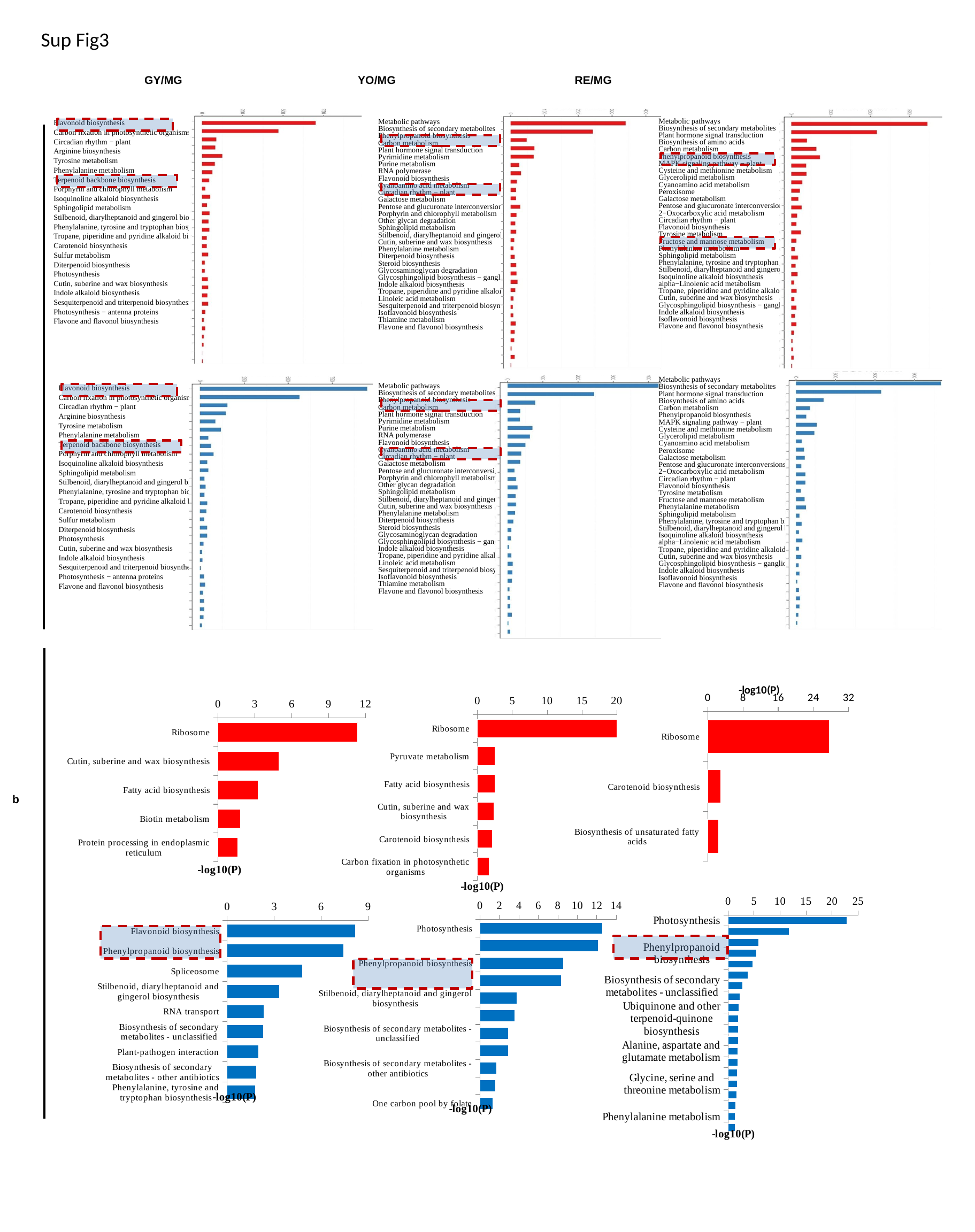

GY/MG YO/MG RE/MG
Flavonoid biosynthesis
Carbon fixation in photosynthetic organisms
Circadian rhythm − plant
Arginine biosynthesis
Tyrosine metabolism
Phenylalanine metabolism
Terpenoid backbone biosynthesis
Porphyrin and chlorophyll metabolism
Isoquinoline alkaloid biosynthesis
Sphingolipid metabolism
Stilbenoid, diarylheptanoid and gingerol biosynthesis
Phenylalanine, tyrosine and tryptophan biosynthesis
Tropane, piperidine and pyridine alkaloid biosynthesis
Carotenoid biosynthesis
Sulfur metabolism
Diterpenoid biosynthesis
Photosynthesis
Cutin, suberine and wax biosynthesis
Indole alkaloid biosynthesis
Sesquiterpenoid and triterpenoid biosynthesis
Photosynthesis − antenna proteins
Flavone and flavonol biosynthesis
Flavonoid biosynthesis
Carbon fixation in photosynthetic organisms
Circadian rhythm − plant
Arginine biosynthesis
Tyrosine metabolism
Phenylalanine metabolism
Terpenoid backbone biosynthesis
Porphyrin and chlorophyll metabolism
Isoquinoline alkaloid biosynthesis
Sphingolipid metabolism
Stilbenoid, diarylheptanoid and gingerol biosynthesis
Phenylalanine, tyrosine and tryptophan biosynthesis
Tropane, piperidine and pyridine alkaloid biosynthesis
Carotenoid biosynthesis
Sulfur metabolism
Diterpenoid biosynthesis
Photosynthesis
Cutin, suberine and wax biosynthesis
Indole alkaloid biosynthesis
Sesquiterpenoid and triterpenoid biosynthesis
Photosynthesis − antenna proteins
Flavone and flavonol biosynthesis
Metabolic pathways
Biosynthesis of secondary metabolites
Plant hormone signal transduction
Biosynthesis of amino acids
Carbon metabolism
Phenylpropanoid biosynthesis
MAPK signaling pathway − plant
Cysteine and methionine metabolism
Glycerolipid metabolism
Cyanoamino acid metabolism
Peroxisome
Galactose metabolism
Pentose and glucuronate interconversions
2−Oxocarboxylic acid metabolism
Circadian rhythm − plant
Flavonoid biosynthesis
Tyrosine metabolism
Fructose and mannose metabolism
Phenylalanine metabolism
Sphingolipid metabolism
Phenylalanine, tyrosine and tryptophan biosynthesis
Stilbenoid, diarylheptanoid and gingerol biosynthesis
Isoquinoline alkaloid biosynthesis
alpha−Linolenic acid metabolism
Tropane, piperidine and pyridine alkaloid biosynthesis
Cutin, suberine and wax biosynthesis
Glycosphingolipid biosynthesis − ganglio series
Indole alkaloid biosynthesis
Isoflavonoid biosynthesis
Flavone and flavonol biosynthesis
Metabolic pathways
Biosynthesis of secondary metabolites
Plant hormone signal transduction
Biosynthesis of amino acids
Carbon metabolism
Phenylpropanoid biosynthesis
MAPK signaling pathway − plant
Cysteine and methionine metabolism
Glycerolipid metabolism
Cyanoamino acid metabolism
Peroxisome
Galactose metabolism
Pentose and glucuronate interconversions
2−Oxocarboxylic acid metabolism
Circadian rhythm − plant
Flavonoid biosynthesis
Tyrosine metabolism
Fructose and mannose metabolism
Phenylalanine metabolism
Sphingolipid metabolism
Phenylalanine, tyrosine and tryptophan biosynthesis
Stilbenoid, diarylheptanoid and gingerol biosynthesis
Isoquinoline alkaloid biosynthesis
alpha−Linolenic acid metabolism
Tropane, piperidine and pyridine alkaloid biosynthesis
Cutin, suberine and wax biosynthesis
Glycosphingolipid biosynthesis − ganglio series
Indole alkaloid biosynthesis
Isoflavonoid biosynthesis
Flavone and flavonol biosynthesis
Metabolic pathways
Biosynthesis of secondary metabolites
Phenylpropanoid biosynthesis
Carbon metabolism
Plant hormone signal transduction
Pyrimidine metabolism
Purine metabolism
RNA polymerase
Flavonoid biosynthesis
Cyanoamino acid metabolism
Circadian rhythm − plant
Galactose metabolism
Pentose and glucuronate interconversions
Porphyrin and chlorophyll metabolism
Other glycan degradation
Sphingolipid metabolism
Stilbenoid, diarylheptanoid and gingerol biosynthesis
Cutin, suberine and wax biosynthesis
Phenylalanine metabolism
Diterpenoid biosynthesis
Steroid biosynthesis
Glycosaminoglycan degradation
Glycosphingolipid biosynthesis − ganglio series
Indole alkaloid biosynthesis
Tropane, piperidine and pyridine alkaloid biosynthesis
Linoleic acid metabolism
Sesquiterpenoid and triterpenoid biosynthesis
Isoflavonoid biosynthesis
Thiamine metabolism
Flavone and flavonol biosynthesis
Metabolic pathways
Biosynthesis of secondary metabolites
Phenylpropanoid biosynthesis
Carbon metabolism
Plant hormone signal transduction
Pyrimidine metabolism
Purine metabolism
RNA polymerase
Flavonoid biosynthesis
Cyanoamino acid metabolism
Circadian rhythm − plant
Galactose metabolism
Pentose and glucuronate interconversions
Porphyrin and chlorophyll metabolism
Other glycan degradation
Sphingolipid metabolism
Stilbenoid, diarylheptanoid and gingerol biosynthesis
Cutin, suberine and wax biosynthesis
Phenylalanine metabolism
Diterpenoid biosynthesis
Steroid biosynthesis
Glycosaminoglycan degradation
Glycosphingolipid biosynthesis − ganglio series
Indole alkaloid biosynthesis
Tropane, piperidine and pyridine alkaloid biosynthesis
Linoleic acid metabolism
Sesquiterpenoid and triterpenoid biosynthesis
Isoflavonoid biosynthesis
Thiamine metabolism
Flavone and flavonol biosynthesis
### Chart
| Category | |
|---|---|
| Ribosome | 20.1 |
| Pyruvate metabolism | 2.53 |
| Fatty acid biosynthesis | 2.49 |
| Cutin, suberine and wax biosynthesis | 2.37 |
| Carotenoid biosynthesis | 2.14 |
| Carbon fixation in photosynthetic organisms | 1.66 |
### Chart
| Category | |
|---|---|
| Ribosome | 27.66 |
| Carotenoid biosynthesis | 2.95 |
| Biosynthesis of unsaturated fatty acids | 2.48 |
### Chart
| Category | |
|---|---|
| Ribosome | 11.36 |
| Cutin, suberine and wax biosynthesis | 4.97 |
| Fatty acid biosynthesis | 3.26 |
| Biotin metabolism | 1.84 |
| Protein processing in endoplasmic reticulum | 1.61 |
### Chart
| Category | |
|---|---|
| Photosynthesis | 22.85 |
| Photosynthesis - antenna proteins | 11.76 |
| Flavonoid biosynthesis | 5.88 |
| Phenylpropanoid biosynthesis | 5.44 |
| Phenylalanine, tyrosine and tryptophan biosynthesis | 4.74 |
| Linoleic acid metabolism | 3.8 |
| Biosynthesis of secondary metabolites - unclassified | 2.78 |
| Glutathione metabolism | 2.26 |
| Carbon fixation in photosynthetic organisms | 2.1 |
| Ubiquinone and other terpenoid-quinone biosynthesis | 2.0 |
| Stilbenoid, diarylheptanoid and gingerol biosynthesis | 1.97 |
| Glyoxylate and dicarboxylate metabolism | 1.94 |
| Alanine, aspartate and glutamate metabolism | 1.91 |
| Oxidative phosphorylation | 1.84 |
| alpha-Linolenic acid metabolism | 1.75 |
| Glycine, serine and threonine metabolism | 1.75 |
| Biosynthesis of secondary metabolites - other antibiotics | 1.64 |
| Tropane, piperidine and pyridine alkaloid biosynthesis | 1.46 |
| Phenylalanine metabolism | 1.39 |
| Nitrogen metabolism | 1.38 |
### Chart
| Category | |
|---|---|
| Photosynthesis | 12.58 |
| Photosynthesis - antenna proteins | 12.12 |
| Phenylpropanoid biosynthesis | 8.57 |
| Flavonoid biosynthesis | 8.35 |
| Stilbenoid, diarylheptanoid and gingerol biosynthesis | 3.78 |
| Phenylalanine, tyrosine and tryptophan biosynthesis | 3.6 |
| Biosynthesis of secondary metabolites - unclassified | 2.9 |
| Linoleic acid metabolism | 2.9 |
| Biosynthesis of secondary metabolites - other antibiotics | 1.7 |
| Ubiquinone and other terpenoid-quinone biosynthesis | 1.58 |
| One carbon pool by folate | 1.31 |
### Chart
| Category | |
|---|---|
| Flavonoid biosynthesis | 8.2 |
| Phenylpropanoid biosynthesis | 7.45 |
| Spliceosome | 4.81 |
| Stilbenoid, diarylheptanoid and gingerol biosynthesis | 3.34 |
| RNA transport | 2.34 |
| Biosynthesis of secondary metabolites - unclassified | 2.3 |
| Plant-pathogen interaction | 2.02 |
| Biosynthesis of secondary metabolites - other antibiotics | 1.86 |
| Phenylalanine, tyrosine and tryptophan biosynthesis | 1.8 |b
Sup Fig3
