# Supplementary material for: Transcriptomic, Proteomic and Metabolomic Analysis of Flavonoid Biosynthesis During Fruit Maturation in Rubus chingii Hu
Source: Front Plant Sci. 2021 Aug 10;12:706667. doi: 10.3389/fpls.2021.706667 (PMC8384110; doi:10.3389/fpls.2021.706667)
Supplement: Supplementary Table 1 — Primers for RT-qPCR analysis. [file Presentation_4.PPTX]

## Slide 1
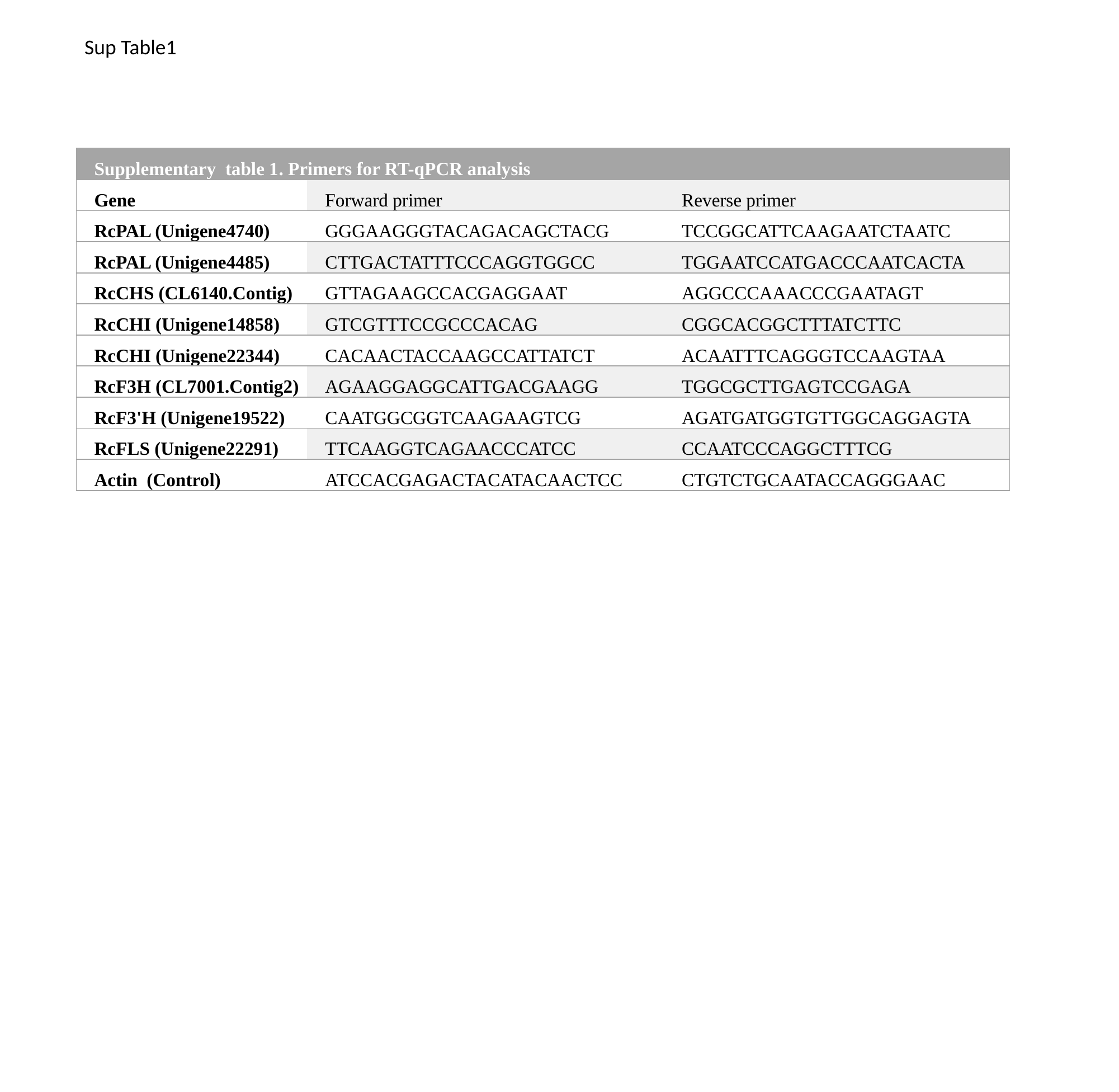

Sup Table1
| Supplementary table 1. Primers for RT-qPCR analysis | | |
| --- | --- | --- |
| Gene | Forward primer | Reverse primer |
| RcPAL (Unigene4740) | GGGAAGGGTACAGACAGCTACG | TCCGGCATTCAAGAATCTAATC |
| RcPAL (Unigene4485) | CTTGACTATTTCCCAGGTGGCC | TGGAATCCATGACCCAATCACTA |
| RcCHS (CL6140.Contig) | GTTAGAAGCCACGAGGAAT | AGGCCCAAACCCGAATAGT |
| RcCHI (Unigene14858) | GTCGTTTCCGCCCACAG | CGGCACGGCTTTATCTTC |
| RcCHI (Unigene22344) | CACAACTACCAAGCCATTATCT | ACAATTTCAGGGTCCAAGTAA |
| RcF3H (CL7001.Contig2) | AGAAGGAGGCATTGACGAAGG | TGGCGCTTGAGTCCGAGA |
| RcF3'H (Unigene19522) | CAATGGCGGTCAAGAAGTCG | AGATGATGGTGTTGGCAGGAGTA |
| RcFLS (Unigene22291) | TTCAAGGTCAGAACCCATCC | CCAATCCCAGGCTTTCG |
| Actin (Control) | ATCCACGAGACTACATACAACTCC | CTGTCTGCAATACCAGGGAAC |
